# Tumor Endothelial Cell-Specific Drug Delivery System Using Apelin-Conjugated Liposomes

**DOI:** 10.1371/journal.pone.0065499

**Published:** 2013-06-14

**Authors:** Hiroki Kawahara, Hisamichi Naito, Kazuhiro Takara, Taku Wakabayashi, Hiroyasu Kidoya, Nobuyuki Takakura

**Affiliations:** 1 Department of Signal Transduction, Research Institute for Microbial Diseases, Osaka University, Suita, Osaka, Japan; 2 Japan Science and Technology Agency (JST), CREST, Tokyo, Japan; Osaka University Graduate School of Medicine, Japan

## Abstract

**Background:**

A drug delivery system specifically targeting endothelial cells (ECs) in tumors is required to prevent normal blood vessels from being damaged by angiogenesis inhibitors. The purpose of this study was to investigate whether apelin, a ligand for APJ expressed in ECs when angiogenesis is taking place, can be used for targeting drug delivery to ECs in tumors.

**Methods and Results:**

Uptake of apelin via APJ stably expressed in NIH-3T3 cells was investigated using TAMRA (fluorescent probe)-conjugated apelin. Both long and short forms of apelin (apelin 36 and apelin 13) were taken up, the latter more effectively. To improve efficacy of apelin- liposome conjugates, we introduced cysteine, with its sulfhydryl group, to the C terminus of apelin 13, resulting in the generation of apelin 14. In turn, apelin 14 was conjugated to rhodamine-encapsulating liposomes and administered to tumor-bearing mice. In the tumor microenvironment, we confirmed that liposomes were incorporated into the cytoplasm of ECs. In contrast, apelin non-conjugated liposomes were rarely found in the cytoplasm of ECs. Moreover, non-specific uptake of apelin-conjugated liposomes was rarely detected in other normal organs.

**Conclusions:**

ECs in normal organs express little APJ; however, upon hypoxic stimulation, such as in tumors, ECs start to express APJ. The present study suggests that apelin could represent a suitable tool to effectively deliver drugs specifically to ECs within tumors.

## Introduction

There are two ways in which blood vessels form [1]. The first is de novo vascular formation, so-called vasculogenesis, in which endothelial cells (ECs) develop in situ and form tubes; the EC tube is then stabilized by the recruitment and adhesion of mural cells to ECs. The other process is angiogenesis, in which a new branch of a blood vessel develops from preexisting vessels by sprouting or intussusception. New blood vessel formation in the adult usually occurs by this process. Diseases such as cancer, retinopathy, and those with a component of chronic inflammation are categorized as vascular diseases because the degree of angiogenesis correlates with disease progression. In the steady state, angiogenesis is not induced except under highly specific conditions such as ovulation during the estrus cycle. It has therefore been considered that targeting angiogenesis is a promising approach for treating vascular diseases with minimum side effects [2].

Thus far, many molecules regulating blood vessel formation have been isolated [3]–[5]. Of these, vascular endothelial growth factor (VEGF) active in development, tube formation and proliferation of ECs, and platelet-derived growth factor (PDGF) and angiopoietin (Ang), important for interactions between ECs and mural cells, it is especially VEGF and its cognate receptors that have been targeted in anti-angiogenesis therapy [6]–[8]. However, although angiogenesis is not induced in the steady state, factors involved in angiogenesis also play critical roles in maintaining blood vessel integrity [9]–[11]. Therefore, it has been reported that high doses of angiogenesis inhibitors can have side effects resulting in disruption of normal blood vessels [12]. This suggests that drug delivery specifically to vascular cells in which angiogenesis is taking place is required for disruption of focal blood vessels without damaging normal vessels.

The receptor tyrosine kinase Tie2, expressed on ECs, induces homotypic cell adhesion between ECs and heterotypic EC- mural cell adhesion on its ligation with Ang1. This favors the structural stability and/or maturation of newly-developed blood vessels [13]. Activation of Tie2 also induces enlargement of blood vessels; we have reported that larger blood vessel formation is controlled by the apelin produced from ECs following their stimulation with Ang1 [14]. When angiogenesis is taking place, VEGF induces expression of the apelin receptor, APJ, a G protein-coupled receptor with 7 transmembrane domains, in ECs. APJ stimulation by apelin induces cell-cell assembly of ECs and VE-cadherin expression in ECs, resulting in enlargement of newly-developing blood vessels [14], [15]. ECs start to express APJ during the angiogenesis process; however, APJ expression is not observed in ECs in the steady state. We recently reported that APJ is abundantly expressed by ECs in tumors [16]. Therefore, we speculated that the apelin/APJ system could be utilized for tumor EC-specific drug delivery. Here, we investigated whether apelin-conjugated liposomes can be internalized via APJ in APJ-expressing cells and whether agents encapsulated in these liposomes could be successfully targeted to tumor EC.

## Materials and Methods

### Materials

Plasmid DNA encoding the enhanced green fluorescent protein (pEGFP-N1) was purchased from Clontech Laboratories (Mountain View, CA). 1,2-dioleoyl-3-dimethylammonium-propane (DODAP), cholesterol (chol), 1-Palmitoyl-2-oleoyl-sn-glycero-3-phosphoethanolamine (POPE), N-(Carbonyl-methoxypolyethyleneglycol5000)-1,2-distearoyl-sn-glycero-3-phosphoethanolamine (DSPE-PEG-5000) and N-[(3-Maleimide-1-oxopropyl) aminopropyl polyethyleneglycol-carbamyl] distearoylphosphatidyl-ethanolamine (DSPE-PEG-5000-Mal) were purchased from NOF Corporation (Tokyo, Japan). 1,2-dioleoyl-*sn*-glycero-3-phosphoethanolamine-N-(lissamine rhodamine B sulfonyl) (DOPE-Rho) were purchased from Avanti Polar Lipids (Alabaster, AL). Lipofectamine 2000 was from Invitrogen Life Technologies (Carlsbad, CA). Apelin 13 and apelin 36 were purchased from Bachem (Bubendorf, Switzerland) and Phoenix Pharmaceuticals (Burlingame, CA), respectively. Apelin 14 was synthesized by Scrum (Tokyo, Japan). Apelin 13-TAMRA and apelin 36-TAMRA were synthesized by Invitrogen Life Technologies.

### Mice

Balb/c and C57BL/6NCr mice were purchased from Japan SLC (Shizuoka, Japan). For the tumor cell transplantation model, 1×10^6^ colon 26 (mouse colon cancer) [16] or B16/BL6 (mouse melanoma) [17] cells were inoculated subcutaneously into 8 week-old female mice. Animals were housed in environmentally-controlled rooms of the animal experimentation facility at Osaka University. All experiments were conducted under the applicable laws and guidelines for the care and use of laboratory animals in the Research Institute for Microbial Diseases, Osaka University, approved by the Animal Experiment Committee of the Research Institute for Microbial Disease, Osaka University.

### Plasmid construction and transfection

DNA encoding mouse APJ was inserted into BamH I and Hind III sites of the pEGFP-N1 expression vector. NIH-3T3 cells were cultured at 37°C in a humidified atmosphere of 5% CO_2_, with Dulbecco's Modified Eagle Medium (D-MEM; Sigma-Aldrich, St. Louis, MO) containing 10% FBS. Before transfection, 10^6^ cells/well were plated in 6-well cell culture plates and allowed to adhere overnight. On the day of transfection, cells were washed once in phosphate-buffered saline (PBS), the culture medium was replaced and cells starved with 1 ml/well of Opti-MEM (GIBCO,Grand Island, NY). After incubation for 3 hrs in Opti-MEM, cells were transfected with pEGFP-N1 (mock vector) or pEGFP-N1-APJ using Lipofectamine 2000 (Invitrogen) according to the manufacturer's instructions. After 6 hrs incubation, the culture medium was replaced with 1 ml/well of D-MEM containing 10% FBS. Stable cell lines expressing mouse APJ-GFP were selected by culture in medium containing 300 µg/ml G418 (GIBCO).

### Western blotting

Cells were washed with ice-cold PBS and lysed with RIPA buffer (50 mM Tris–HCl pH 7.5, 150 mM NaCl, 1% NP-40, 0.5% sodium deoxycholate, 0.1% SDS) for the whole lysate and TM-PEK (Merck Chemicals, Frankfurter, Germany) for membrane and cytosol samples, according to the manufacturer's instructions. Proteins electrophoretically separated using 7.5% SDS gels were transferred to PVDF membranes (GE Healthcare, Amersham Place, UK) by a wet blotting procedure (100 V, 350 mA, 60 min). The membrane was blocked with 5% skim milk/TBST for 60 min, subsequently incubated with anti-GFP (MBL, Nagoya, Japan), anti-β-actin (Sigma-Aldrich), and anti-flotillin-1 (BD Biosciences, San Diego, CA) antibodies (Abs) and processed for chemiluminescence detection with ECL solution (GE Health Care).

### Flow cytometry

Mouse APJ-GFP-expressing NIH-3T3 cells (5×10^5^ cells/well) were seeded onto 12 well plates and cultured overnight. Apelin-TAMRA was added to cells at 1 mM and incubated for 2 hrs. After incubation, cells were rinsed, trypsinized, and analysed using FACS Calibur (BD Bioscience).

### Liposome preparation and characterization

DODAP, chol, POPE, DSPE-PEG-5000 and DOPE-Rho at a molar ratio of 202∶101∶101∶91∶5 were dissolved in ethanol and chloroform at a ratio of 1∶1 (v/v). For apelin-conjugated liposomes, DSPE-PEG-5000-Mal replaced half the DSPE-PEG-5000. A thin lipid film was formed in a glass test tube using a rotary evaporator (EYELA, Tokyo, Japan). The lipid film was hydrated in PBS for 10 min and sonicated for 3 min. Apelin 14 was added to each resulting liposome preparation and incubated at room temperature overnight. After incubation, liposomes were thrice filtered using Amicon Ultra Centrifugal Filter Units, 30 kDa (Millipore, Billerica, MA). After filtration, the cholesterol concentration in liposomes was measured using the Cholesterol E-test WAKO (Wako Pure Chemical Industries, Osaka, Japan) for total lipid concentration. The particle size distribution, average particle size and zeta-potential were measured by Katayama Chemical, Ltd. (Osaka, Japan). The reaction of apelin 14 with DSPE-PEG-5000-Mal was analysed using MALDI-QIT-TOF AXIMA Performance by Shimadzu Corporation (Kyoto, Japan).

### Time-lapse analysis and confocal microscopy

Before time-lapse analysis, cells seeded onto glass-bottom dishes were incubated with Minimum Essential Medium Eagle (MEM; Sigma-Aldrich) containing 0.1% BSA and 20 mM HEPES for 24 hrs. After observation of cells for 5 min, apelin was added to 100 nM and observed for 25 min. Time-lapse analysis was performed using Leica DMI 6000B for confocal microscopy (Werzlar, Germany). Cells seeded on poly-L-lysine–coated Millicell EZ slides (Millipore) were incubated with MEM containing 0.1% BSA and 20 mM HEPES for 24 hrs. Liposomes were added to cells at 1 mM and incubated for 30 min. Thereafter, the cells were rinsed, fixed for 10 min in 4% paraformaldehyde-PBS (pH 7.5) and washed with PBS. The cells were observed under Leica DM5500B equipped with HCX PL FLVOTAR 5/0.15 and HCX PL FLVOTAR 10_/0.15 dry objective lenses. All images were processed with Adobe Photoshop CS5 Extended software (Adobe Systems, San Jose, CA).

### Intra-tumoral distribution of liposomes, and immunofluorescence

1 mM/mouse of liposomes were administered through the tail vein. Tissue fixation and immunohistological analyses of tissue sections was carried out as described previously [18]. Briefly, the fixed specimens were embedded in OCT compound and sectioned at 8 µm. For immunofluorescence, anti-CD31 monoclonal antibody (BD Biosciences) was used for staining and anti-rat IgG Alexa Fluor 488 (Invitrogen) as the secondary antibody. Cell nuclei were visualized with Hoechst dye (Sigma). Samples were visualized using the Leica DM5500B. All images were processed with Adobe Photoshop CS5 Extended software (Adobe Systems).

## Results

### Internalization of APJ and apelin into the cytoplasm following activation with apelin 13 and apelin 36

**Figure 1:**
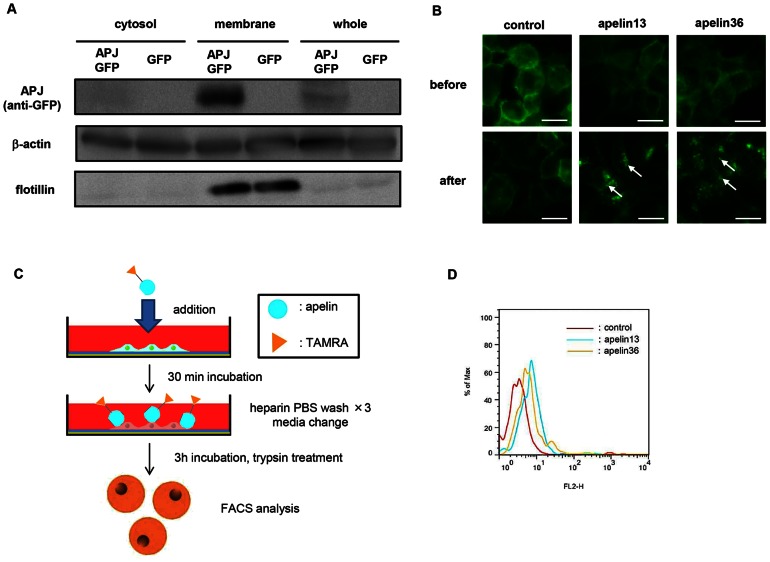
Internalization of apelin via cell surface APJ. (**A**) Western blot analysis of mouse APJ-GFP fusion protein on NIH-3T3 cells. Anti-GFP antibody was used to detect APJ-GFP. Flotillin is the positive control for membrane proteins on NIH-3T3 cells. β-actin was used as the internal control. (**B**) Internalization of mouse APJ-GFP with apelin. NIH3T3-APJ-EGFP cells were incubated in the presence or absence of apelin 13 or apelin 36. White arrows indicate internalized APJ. Bar indicates 20 µm. (**C**) Scheme for apelin internalization analysis. NIH3T3-APJ-EGFP cells were incubated with apelin (13 or 36)-TAMRA for 3 hrs, washed with heparin PBS to remove non-specifically bound apelin-TAMRA from the cell membrane. Cell membrane proteins were trypsinized and cells were analyzed by flow cytometry. (**D**) APJ-mediated uptake of apelin-TAMRAs by flow cytometry.

As far as we have been able to establish, EC lines such as bEnd3 (brain microvessel -derived ECs) or MS1 (mouse pancreatic islet-derived ECs) do not express APJ. Moreover, stable APJ expression in primary ECs such as human umbilical vein ECs (HUVECs) was also not observed. Therefore, we generated stable transformants of NIH-3T3 cells expressing APJ (NIH3T3-APJ-EGFP) by transfection of APJ-EGFP fusion gene expression plasmids. As observed in western blotting, APJ (EGFP) expression in NIH3T3-APJ-EGFP was found mostly in the membrane, but not in the cytosolic fraction of these cells (**Figure 1A**). In living cells, APJ-EGFP expression was observed in the cell membrane (**Figure 1B**). After stimulation with apelin 13 or apelin 36 (C terminal 13 or 36 amino acids derived from the 77 amino acid–long pre-pro-apelin protein, respectively), APJ-EGFP was internalized into the cytoplasm. The degree of internalization of APJ was identical for both apelin 13 or apelin 36 stimulation (**Figure 1B**).

It has been reported that the APJ-mediated cellular uptake pathway for apelin is different for apelin 13 and apelin 36 stimulation and that APJ was recycled upon stimulation with apelin 13 but not apelin 36 using HEK193 cells [19]. However, our result using NIH3T3 cells was completely different (data not shown). It is suggested that β-arrestin is required for apelin 13 mediated recycle of APJ. As far as we examined, NIH3T3 did not express β-arrestin (data not shown), indicating that another mechanism is involved in apelin 36 mediated recycle of APJ. Therefore, we compared which apelin form is optimal for delivery of liposome-conjugated apelin via APJ (**Figure 1C, D**). TAMRA (fluorescent dye)-conjugated apelin 13 or apelin 36 was added to NIH3T3-APJ-EGFP cells and incubated for 30 minutes. After removal of free apelin by washing, cells were incubated for 3 hours, washed again, and cell surface proteins removed by trypsin treatment.

TAMRA fluorescence intensity in cells was analyzed by flow cytometry, showing that more apelin 13 than apelin 36 was taken up (**Figure 1D**). This suggests that apelin 13 would be more efficient for drug delivery.

### Generation of apelin-conjugated liposomes

**Figure 2:**
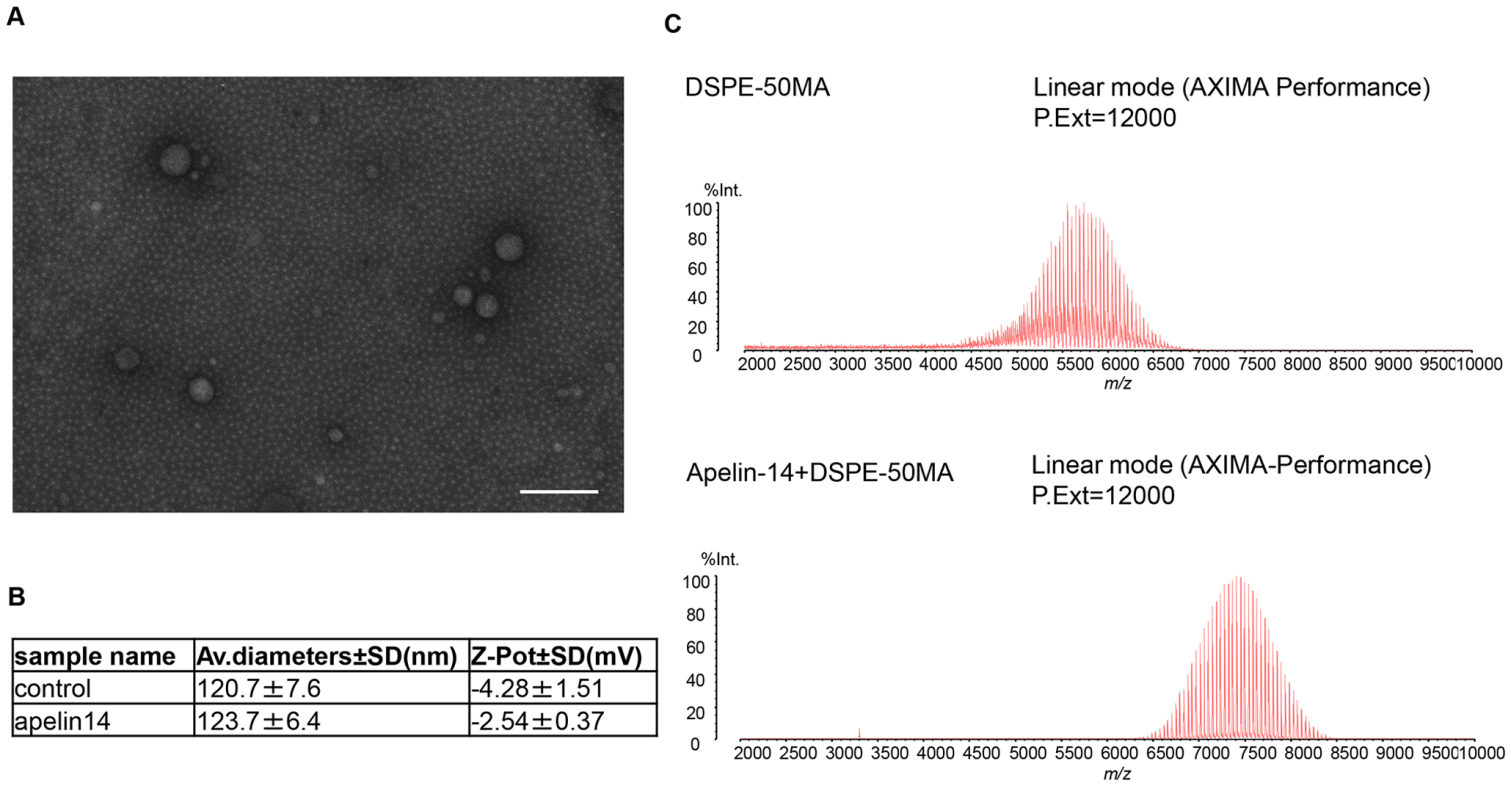
Liposome characterization. (**A**) Electron micrograph of liposomes. Bar indicates 200 nm. (**B**) Average particle size and zeta-potential as determined by dynamic light scattering. (**C**) Mass spectrometry of DSPE-50MA (DSPE-PEG-5000-Mal) and apelin 14 with DSPE-50MA.

We confirmed by electron microscopy that the size of the liposomes generated was <100 nm (**Figure 2A**). For apelin conjugation to liposomes, there are few functional groups in the apelin 13 amino acids available for the reaction. Therefore, we added cysteine, having sulfhydryl groups to the C terminus of apelin 13, resulting in generation of apelin 14. Synthesized apelin 14 labeled with TAMRA also induced internalization of APJ in the same way as seen in Figure 1B (data not shown). Moreover, the binding efficacy of apelin 14 to APJ was equivalent to that of apelin 13 (data not shown). Therefore, we prepared apelin 14-conjugated liposomes in which N-terminal maleimide base-coupled polyethylene glycol (PEG) was initially conjugated to the liposome (DSPE-50MA) and apelin 14 then bound to DSPE-50MA (Apelin14-DSPE-50MA). The average size of DSPE-50MA and Apelin14-DSPE-50MA was 120∼124 nm (**Figure 2B**). Apelin 14 conjugation slightly increased liposome size from 120 nm to 123 nm. The average zeta potential of DSPE-50MA and Apelin14-DSPE-50MA was −4.28 mV and −2.54 mV, respectively; however, this difference is not large and based on these values, we confirmed that these liposomes were neutral.

To confirm conjugation of apelin 14 to DSPE-50MA, liposomes were analyzed by mass spectrometry. As shown in **Figure 2C**, there is only one peak derived from DSPE-50MA and Apelin14-DSPE-50MA. We confirmed that the Apelin14-DSPE-50MA peak was shifted to the right of DSPE-50MA, by an amount corresponding to the molecular weight of apelin 14 (Mw = 1653). Therefore, this suggested that apelin 14 was conjugated to DSPE-50MA

### Incorporation of apelin-conjugated liposomes into APJ-expressing cells

**Figure 3:**
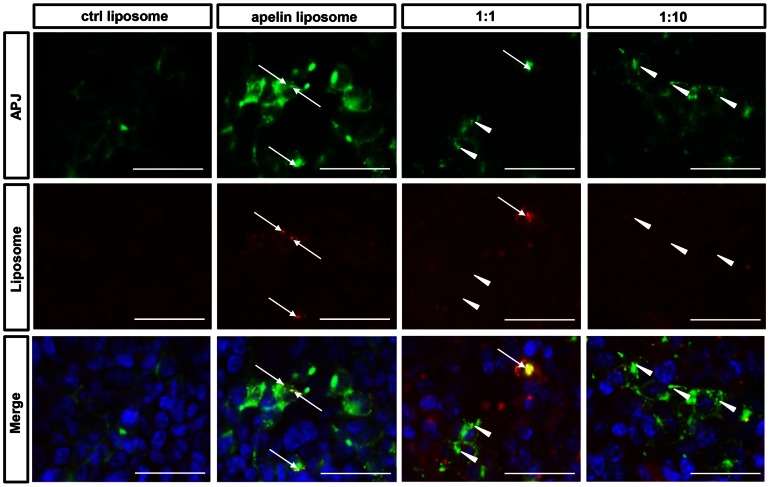
Uptake of apelin-conjugated liposomes via APJ. Fluorescence microscopic images of NIH3T3-APJ-EGFP incubated with control- (left) or apelin14-liposomes (right three lines). Competition analysis using free liposomes and apelin14-liposomes (right two lines). Free apelin was added at a molar ratio of 1∶1 or 1∶10 (apelin14-liposomes∶free apelin). Arrows in the second and third lines indicate examples showing merged APJ with rhodamine encapsulated in liposomes. Arrow heads in the third and fourth lines indicate internalized APJ but are not merged with rhodamine. Bars indicate 50 µm.

Next, we investigated whether Apelin14-DSPE-50MA (henceforth referred to as Apelin14-Liposomes) is internalized into the cytoplasm of NIH3T3-APJ-EGFP cells (**Figure 3**). The fluorescent dye rhodamine was included in the liposomes; we used apelin14-Liposomes and as a negative control, apelin non-conjugated DSPE-50MA (control liposomes). Following stimulation with control liposomes, APJ remained on the cell surface and no rhodamine was observed in the cytoplasm. In contrast, when cells were stimulated with apelin14-Liposomes, APJ internalized into the cytoplasm and showed a vesicle-like appearance. Thus, we confirmed the localization of rhodamine in the cytoplasm, suggesting that the liposomes were taken up. There were also cells with inclusions having a vesicle-like appearance by APJ-EGFP clustering but they did not manifest any rhodamine uptake in the cytoplasm. This might be caused by insufficient liposome uptake, although APJ was stimulated by apelin.

To assess whether liposome internalization was mediated by APJ, we performed competition analysis using apelin 14 and apelin14-Liposomes. The addition of higher doses of free apelin together with a fixed dose of apelin14-Liposomes resulted in similar levels of APJ internalization but rhodamine uptake was suppressed (**Figure 3**). This strongly suggests that apelin14-Liposomes uptake is mediated specifically by APJ.

### Distribution of apelin14-Liposomes following administration into tumor-bearing mice

**Figure 4:**
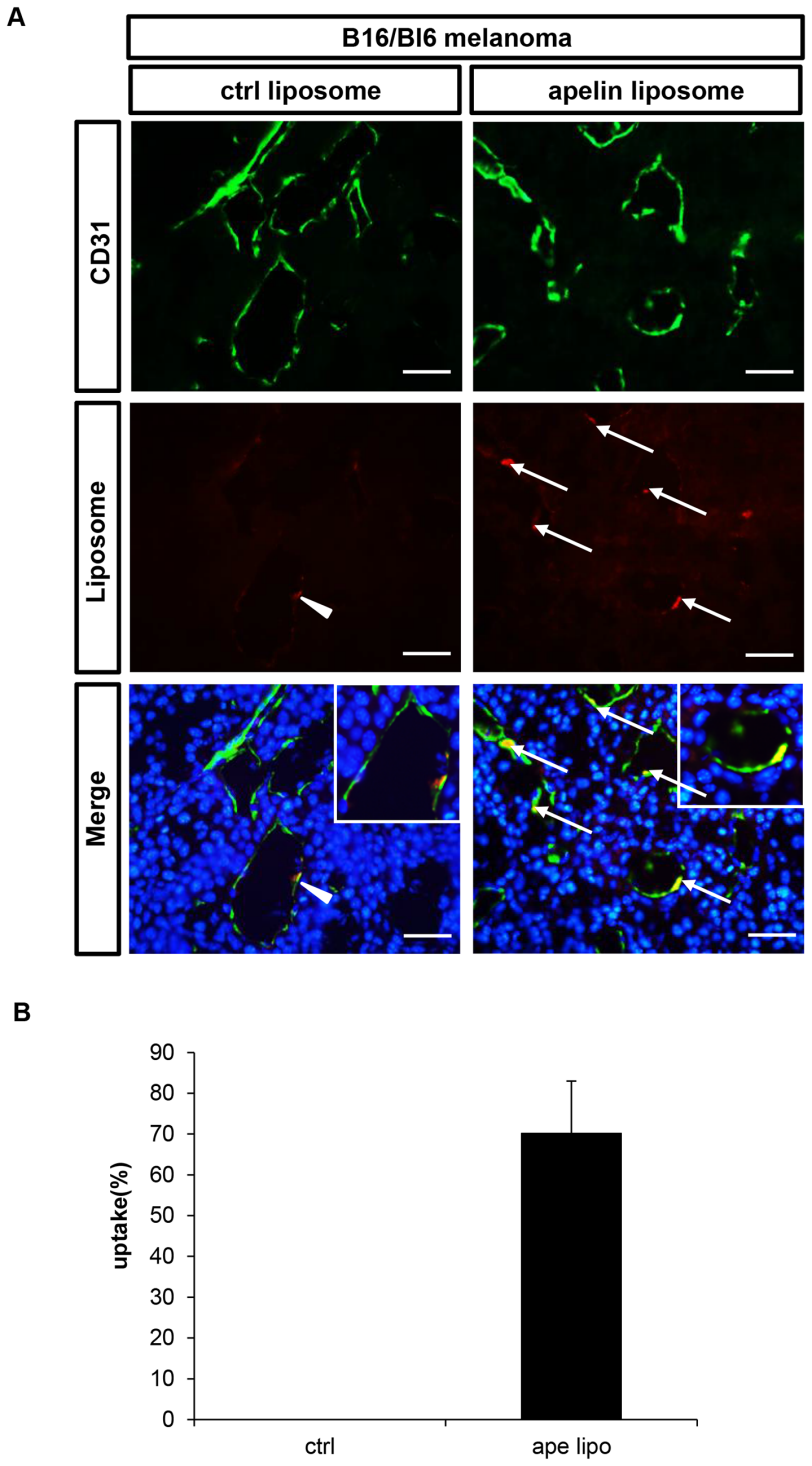
Uptake of apelin14-liposomes into ECs of B16/BL6 tumors. (**A**) Fluorescence microscopic images of B16/BL6 tumor sections from mice administered control liposomes (left) or apelin14-liposomes (right). CD31 (green), rhodamine encapsulated in liposomes (red), and DAPI (blue). Arrowheads indicate liposomes adhering to CD31^+^ ECs but not incorporated into them. Arrows indicate liposomes incorporated into CD31^+^ ECs. Bars indicate 50 µm. (**B**) Ratio of liposomes incorporated into ECs among all liposomes. Five random fields were observed.

We next tested whether apelin14-Liposomes can be specifically taken up by tumor ECs. First, B16/BL6 mouse melanoma cells were inoculated s.c. into recipients; after 10 days we confirmed that tumor mass had grown to a volume of 100 mm^3^. At this point, we injected apelin14-Liposomes and control liposomes intravenously into the tail vein. 24 hours later, we dissected tumors and quantified liposome uptake into tumor ECs histologically. In the case of control liposomes, some Rhodamine was found to be located near CD31-positive ECs in tumors, but not in their cytoplasm as documented in **Figure 4A** by lack of overlap with the green fluorescence of CD31 staining. In contrast, using apelin14-Liposomes, rhodamine colocalized with CD31, suggesting efficient uptake into the EC cytoplasm (**Figure 4A**). ECs incorporating the dye into their cytoplasm after treatment with apelin14-Liposomes was, at 70% of all rhodamine-positive ECs, including cells in which rhodamine was adhering to the cell surface, markedly higher than with the control liposomes (**Figure 4B**).

**Figure 5:**
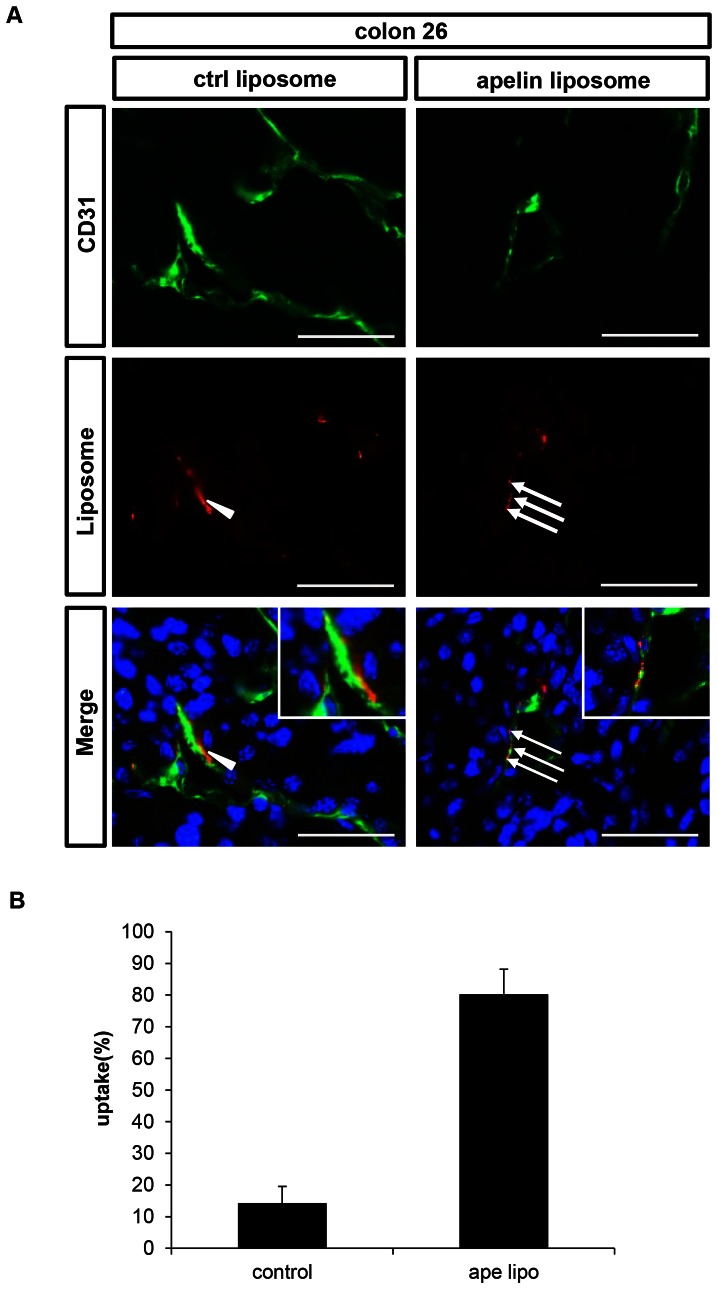
Uptake of apelin14-liposomes into ECs of colon 26 tumors. (**A**) Fluorescence microscopic images of colon 26 tumor sections from mice administered control liposomes (left) or apelin14-liposomes (right). CD31 (green), rhodamine encapsulated in liposomes (red), and DAPI (blue). Arrowheads indicate liposomes adhering to CD31^+^ ECs but not incorporated into them. Arrows indicate liposomes incorporated into CD31^+^ ECs. Bars indicate 50 µm. (**B**) Ratio of liposomes incorporated into ECs among all liposomes. Five random fields were observed.

We also used colon 26, a mouse colon cancer for evaluation of liposome uptake in the same manner as for the B16/BL6 experiments. We injected apelin14-Liposomes or control liposomes i.v through the tail vein after tumor volume had reached 100 mm^3^ after s.c. inoculation. Results were similar to those in the B16/BL6 melanoma system. In brief, control liposomes were again found near CD31-positive ECs in tumors, but not in their cytoplasm (**Figure 5A**), whereas apelin14-Liposomes were efficiently taken up into the cytoplasm (**Figure 5A**). Apelin14-Liposomes were found in the cytoplasm of 80% of all rhodamine-positive ECs (**Figure 5B**).

## Discussion

Here we used apelin to assess whether tumor EC-specific drug delivery is feasible via APJ expressed on the ECs. We found that apelin-conjugated liposomes were specifically taken up by tumor ECs. Moreover, we could not detect non-specific uptake of apelin14-Liposomes in ECs of normal organs such as liver and spleen (data not shown). When APJ positivity in tumor ECs was evaluated, the ratio of APJ expressing vessels in colon26 and B16/BL6 melanoma was approximately 57% and 79%, respectively. However, when the frequency of ECs taking up liposomes was quantified, we found that it was very low in relation to all ECs in the tumor. Because tumor interstitial fluid pressure varies depending on the permeability of the tumor vasculature [20], liposomes do not penetrate into all parts of the tumor equally. Looking only at the tumor areas where Rhodamine vesicles were relatively abundant, as observed in Figure 4A, one can see that approximately 10% of all ECs in the tumor took up apelin14-Liposomes. How to accomplish equal and efficient liposome distribution throughout the tumor microenvironment is a challenge for the future.

In addition to the difficulty related to liposome penetration into the tumor microenvironment, fragility of apelin as a peptide also deserves consideration; it has been suggested that apelin degrades soon after intravenous injection into mice (data not shown). Therefore, instead of apelin, an antibody against APJ may be more useful for ligating this receptor expressed on tumor ECs. Further analysis concerning whether APJ antibody-conjugated liposomes are effectively taken up by ECs is required.

Liposome-encapsulated adriamycin is already in clinical use, and can be effective for tumor inhibition [21]. Adriamycin may inhibit growth of ECs; this would make this liposome appropriate for conjugation with apelin or APJ antibody. However, APJ is also weakly expressed by neuronal cells and cardiomyocytes [22], [23]. Therefore, it may be better to use EC-specific inhibitors such as tyrosine kinase inhibitors for VEGF receptors. It has been suggested that higher doses of VEGF inhibitors may damage healthy blood vessels in normal organs [12]. Therefore, there is a dose limitation for VEGF inhibitors in clinical use when administered systemically. Tumor EC-specific targeting would prevent such side effects. In summary because few tumor EC-specific antigens have been identified, targeting APJ as well as integrin αvβ3 as previously reported, which integrin binding peptide RGD conjugated nanoparticles accumulate and deliver drugs to ECs in tumor effectively, [24], [25] will be useful to disrupt blood vessel formation specifically in tumors.
